# Environmental and Nutritional Effects Regulating Adipose Tissue Function and Metabolism Across Generations

**DOI:** 10.1002/advs.201900275

**Published:** 2019-04-16

**Authors:** Wenfei Sun, Ferdinand von Meyenn, Daria Peleg‐Raibstein, Christian Wolfrum

**Affiliations:** ^1^ Department of Health Science and Technologies ETH Zürich Schorenstrasse 16 Schwerzenbach CH‐8603 Switzerland

**Keywords:** adipose tissue, diabetes, epigenetics, obesity, transgenerational epigenetic inheritance

## Abstract

The unabated rise in obesity prevalence during the last 40 years has spurred substantial interest in understanding the reasons for this epidemic. Studies in mice and humans have demonstrated that obesity is a highly heritable disease; however genetic variations within specific populations have so far not been able to explain this phenomenon to its full extent. Recent work has demonstrated that environmental cues can be sensed by an organism to elicit lasting changes, which in turn can affect systemic energy metabolism by different epigenetic mechanisms such as changes in small noncoding RNA expression, DNA methylation patterns, as well as histone modifications. These changes can directly modulate cellular function in response to environmental cues, however research during the last decade has demonstrated that some of these modifications might be transmitted to subsequent generations, thus modulating energy metabolism of the progeny in an inter‐ as well as transgenerational manner. In this context, adipose tissue has become a focus of research due to its plasticity, which allows the formation of energy storing (white) as well as energy wasting (brown/brite/beige) cells within the same depot. In this Review, the effects of environmental induced obesity with a particular focus on adipose tissue are discussed.

## 1 Introduction

The global epidemic of obesity, which has been subject of debate and research since more than 30 years is still accelerating in its progression. Classified by body mass index (BMI), nearly 40% of all adults are overweight while 13% are considered to be obese.^1^ This poses a burden on life quality and health care costs as obesity is correlated with a host of secondary disorders such as type 2 diabetes, hypertension, dyslipidemia, nonalcoholic fatty liver, coronary heart disease, stroke, osteoarthritis, cancer, and gallbladder disease, to name but a few.^2^ In addition, the obesity epidemic has reached developing children; in this context it was reported that 381 million children under 19 were overweight or obese in 2016.^1^ Given the prevalence for the above‐mentioned secondary complications in conjunction with the fact that obesity at a young age is likely to persist throughout the life,^3^, ^4^ these developments are considered to be the driving force behind the increase in the prevalence of noncommunicable diseases. This in turn poses a major social and medical burden worldwide estimated at two trillion US dollars per year^5^ and accounting for 4 million of death and 120 million disabilities.^6^

On the simplest level, the fundamental cause of obesity is an imbalance in energy intake over expenditure, however, which factors drive this observed energy imbalance and to what extent the contribute to the progression of obesity remains less clear. It is well established that multiple factors are based on changes in food intake and energy metabolism, which is systemically controlled by the central nervous system (CNS) and engages the entire body. Similarly, it is known that both genetic background and environmental factors play an important role in controlling energy homeostasis. Interestingly, several epidemiological studies as well as twin studies demonstrate that obesity is highly heritable. Nevertheless, whole genome sequencing reports have uncovered only a small percentage of inherited BMI due to genetic variance, leaving a large portion of the variance unexplained.^7^ Recent studies in both human and animal models have emphasized the cross‐talk between the environment and genetics supporting the notion that metabolic disease risks could be transmitted across generations in response to different environmental stimuli. This suggests that epigenetic mechanisms are involved in the inheritance of metabolic risks. In contrast to genetic polymorphism, epigenetic modifications that include DNA methylation, histone modifications, and small noncoding RNAs are typically reversible and thus might present targets to stem the increase in obesity.

## 2 Etiology of Obesity

**Figure 1:**
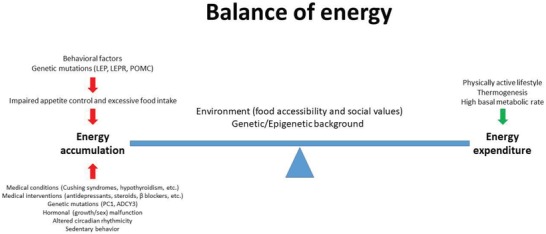
Energy balance: the main determinant of body weight is controlled by multiple factors, which regulate energy accumulation and expenditure.

Understanding the cause of excessive positive energy balance is the first step to develop therapies for obesity. Although a positive energy balance is the most common denominator for excessive fat accumulation, obesity is a heterogeneous condition, which involves behavioral, genetic, and environmental factors as illustrated in **Figure** **1**.

First off, medical conditions such as Cushing syndrome, hypothyroidism, and Prader‐Willi syndrome will lead to obesity.^8^, ^9^, ^10^ Similarly, side effects of certain medications like antidepressants, antipsychotics, steroids, β‐blockers, as well as others have been demonstrated to induce obesity.^11^, ^12^, ^13^ Furthermore, several rare cases of monogenic obesity have been described, which are usually associated with severe early‐onset obesity in conjunction with other endocrine disorders, mainly independent of environmental factors. Such forms of obesity are often due to mutations in genes of the leptin/melanocortin axis involved in food intake regulation [genes of leptin (LEP) and leptin receptor, proopiomelanocortin (POMC), proconvertase 1] or other genes linked to these pathways.^14^ As this Review focuses on epigenetic factors leading to an increased propensity for obesity development, monogenic obesity will not be discussed; an overview can be found here.^15^, ^16^

Important additional driving forces for the development of obesity are the so‐called “behavioral factors.” In this context, dietary preferences and food intake, physical activity, and circadian rhythm have been demonstrated to affect body weight gain. For example, it was reported that people with excessive high sugar and/or high fat food consumption are more likely to be obese and develop secondary complications. Similarly, a sedentary life style has been identified as a risk factor for excessive weight gain.^17^ Epidemiological evidence also suggests that an irregular circadian rhythm or a constant disruption of such a rhythm can promote weight gain and lead to the development of metabolic disorders.^18^, ^19^

**Figure 2:**
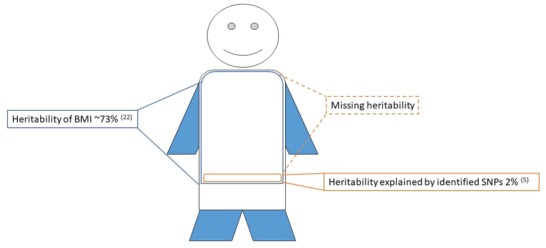
Body weight is highly heritable,^24^, ^25^ however DNA sequence variations identified so far, account for only a small percentage of the observed heritability. This mismatch might potentially be due to epigenetic regulation.

Besides the above‐mentioned factors which have been identified as possible cues to explain the rise in obesity, it is well accepted that inherited genetic variance together with social behavior has a strong impact on the body weight of an individual. Already more than 30 years ago, a twin study demonstrated that the genetic heritability for BMI has a correlation of 0.78 at the age of 20,^20^ and monozygotic twins have twice the concordance rates of overweight than dizygotic twins. Furthermore, a study, which enrolled more than 600 twin sisters, showed that the heritability of waist‐hip ratio is between 0.36 and 0.61.^21^ Genome‐wide association studies (GWAS) have contributed significantly to the identification of single‐nucleotide polymorphisms (SNPs) related to the variability of the BMI. Overall, more than 300 SNPs have been reported in individual studies to be associated with different obesity traits.^22^ A large meta‐analysis based on 46 studies in conjunction with a follow‐up study by the Genetic Investigation of Anthropometric Traits (GIANT) consortium identified more than 30 SNPs, which can be considered robustly associated with BMI.^7^ Interesting to note is that most of these genes are associated with neurol functionality suggesting that regulation of food intake is one important driver for an increased BMI. Notably there have been associations reported of waist–hip ratio and general adiposity with genes linked to adipose tissue function,^22^ suggesting that many genes affecting multiple organ systems contribute to the development of obesity. Nevertheless, even in combination, the identified SNPs can only explain approximately 2% of the variation observed in the BMI.^7^ This mismatch between the heritability estimates and the variation explained through GWAS findings has been coined the “missing heritability,” as illustrated in **Figure** **2**. Taken together, these studies indicate that there is a strong heritable component of obesity and that the interaction of life style and behavior can ultimately predispose an individual toward the development of a positive energy balance, which in the long run can lead to the development of obesity.

In conclusion, genetic variance plays an important role in regulating body weight, however the steep increase in the obesity prevalence during the last three decades is probably due to behavioral and environmental factors,^23^ which might be mediated by various epigenetic mechanisms.

## 3 Adipose Tissue Plasticity and Energy Homeostasis

### 3.1 Adipose Tissue Composition

**Figure 3:**
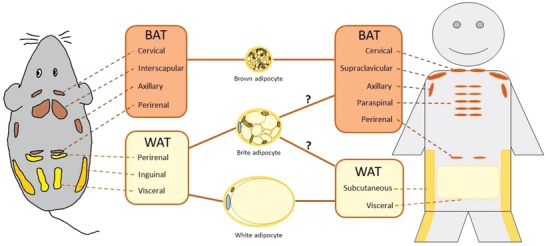
Adipose tissue distribution in humans and mice. Brown adipose tissue composed of either brown or brite/beige adipocytes in humans is mainly localized in cervical, supraclavicular, axillary, paraspinal, and perirenal depots. In mice, brown adipocytes are located in the interscapular depot while brite/beige adipocytes are found interspersed in different depots.

The adipose organ that is expanded in the context of obesity is organized in different depots or tissues, which are located throughout the body. As illustrated in **Figure** **3**, two major tissues compose the adipose organ, namely the brown adipose tissue (BAT) and the white adipose tissue (WAT).^26^ Both are made up by a very heterogeneous cell pool, which can be loosely grouped into two main clusters. The major functional cell pool comprises the mature adipocytes, which represent 20% to 30% of the total cells.^27^ Furthermore, adipose tissue contains the stroma vascular fraction (SVF), which harbors—according to current belief—the major heterogeneity of the adipose tissue and is composed of preadipocytes, mesenchymal stem cells, fibroblasts, macrophages, immune cells, endothelial cells, and vascular progenitors.^26^, ^28^

The main parenchymal cells of the adipose organ are adipocytes, which currently are again loosely grouped into three major cell types. White adipocytes store the energy taken up from the circulation into tightly packed triacylglycerols.^29^ In case of energy deficiency, fatty acids can be rapidly released back into circulation and function as a fuel source for other cells within the organism. Contrary, brown adipocytes, and a less thermogenic efficient‐related population referred to as beige or brite/beige adipocytes (derived from brown‐in‐white adipocytes), dissipate chemical energy in form of heat (thermogenesis) to protect from cold through nonshivering thermogenesis and to counteract energy overload. This unique ability^30^ is mainly enabled through the specific expression of uncoupling protein 1 (UCP1) in mitochondria. Notably, brown and brite/beige adipocytes share some similar morphological, biochemical, and thermogenic characteristics, such as multiple small lipid droplets and a high abundance of mitochondria; however, they possess also various distinct features.^31^ In rodents, classical brown adipocytes are located within distinct areas, such as the interscapular BAT. Brite/beige cells on the other hand are inducible and can be found interspersed within various WAT depots upon cold acclimation, β3‐adrenergic receptor agonist stimulation as well as in response to other stimuli.^27^, ^32^

### 3.2 Adipose Tissue Development

Adipose tissue development is initiated at week 14 of embryonic gestation in humans and can be separated according to Poissonnet et al. into different morphogenic phases.^33^ After the appearance of fat lobules typical vacuolated fat cells appear. After the week 23 of gestation, the total number of fat lobules remains approximately constant and thus adipose tissue expansion from week 23 to 29 is mainly determined by an increase in the size of the lobules. These data suggest that the most critical window for affecting human adipose tissue function during development is week 23 to 29 of gestation. Nevertheless, it should be noted that even in adulthood adipose tissue turnover is observed.^34^

In rodents, BAT is formed at embryonic day 15.5,^35^ while WAT only starts to accumulate lipids after birth,^36^ suggesting that the critical time window for affecting adipose tissue development might be different in mice versus humans. Generally, lipid containing adipocytes are differentiated from multipotent, self‐replenishing precursor cells, through adipogenesis, which occurs at prenatal and postnatal stages and is required for adipocytes formation and turn over.^34^ The origin of adipogenesis has been intensively studied,^37^, ^38^, ^39^ but the exact precursor cell type that gives rise to adipocytes has not been identified.^40^, ^41^, ^42^ At the cellular level, adipogenesis is regulated by the extracellular matrix^43^ and the neighboring cells.^44^, ^45^ Meanwhile, this process is orchestrated by a complex of transcription factors^46^, ^47^ and numerous hormonal molecules^48^, ^49^ at the molecular level.

### 3.3 Adipose Tissue Hypertrophy and Hyperplasia

The adipose organ is extremely plastic as it can change in size in response to environmental cues such as energy overload/deficiency or changes in temperature. This is attributed to two mechanisms, namely hypertrophy (increase in adipocyte size) and hyperplasia (increase in adipocyte number). It was reported that upon high fat diet (HFD) feeding, subcutaneous WAT increases its size mainly by hypertrophy while visceral WAT size increase is at least in part due to hyperplasia.^50^ This demonstrates that environmental stimuli can differentially affect the recruitment of new cells based on the anatomical location of the adipose tissue and further underlines the plasticity of this organ.

The balance between hypertrophy and hyperplasia is strongly associated with the metabolic state of obesity. Smaller adipocytes retain their insulin sensitivity, while hypertrophic adipocytes are more prone to become insulin resistant and to secret proinflammatory cytokines,^51^ such as tumor necrosis factor alpha (TNFα) and interleukin 6 (IL‐6), which could potentially induce insulin resistance in other tissues.^52^ Furthermore, adipose tissue with hypertrophic adipocytes is less vascularized and hypoxic, which can lead to elevated levels of angiogenic factors, which in turn can accelerate adipose tissue fibrosis and inflammation.^53^, ^54^, ^55^

Contrastingly, hyperplasic expansion of adipose tissue is correlated with a beneficial metabolic profile. Many clinical studies have reported that certain obese individuals (BMI > 30) are metabolically healthy, with normal Homeostatic Model Assessment for Insulin Resistance (HOMA‐IR), glucose, and lipid levels. A frequent and distinguishable feature of these metabolic healthy obese patients is the more numerous and smaller adipocytes, compared to metabolic impaired obese individuals.^56^, ^57^ In rodent models, artificially inducing de novo adipocyte formation by pharmacological^58^, ^59^ or genetic approaches^60^, ^61^ leads to an improved insulin sensitivity and enhanced energy storage capacity. These findings suggest that de novo differentiation of adipocytes from preadipocytes exerts a beneficial effect to protect from obesity induced metabolic unbalance.

### 3.4 Depot‐Specific Differences of the Adipose Organ

Another example highlighting the importance of adipose tissue plasticity and its dependency on the environment is the large differences in size that are observed in a depot‐specific manner due to environmental cues.^62^ For example, it was reported that increased amounts of subcutaneous WAT compared to visceral WAT are correlated with a healthier phenotype. As subcutaneous and visceral WAT show various depot‐dependent differences, such as developmental lineage,^63^ gene expression,^64^ and metabolic characteristics,^65^ it is possible that either intrinsic tissue function or anatomic location contribute to the observed differences. In this context, it was shown that adipose tissue distribution and expandability are influenced by intrinsic factors like microenvironment, gender, and age.^66^, ^67^ Male and female adipose tissue shows various differences including amount, distribution, metabolic capability, and function.^68^ Women in general have a higher percentage of total body fat than men, however this seems not to be connected to an increased risk for metabolic diseases, which might be due to an altered functionality or anatomical location.^69^ This differences in functionality is believed to be influenced mainly by the microenvironment in each depot as well as sex hormones such as estrogens,^67^ it should be noted however that cellular heterogeneity has not been studied extensively due to technical challenges.

Also preadipocytes seem to be regulated in a sex and depot‐specific manner and thus can be expected to influence distribution and function.^67^ During pregnancy and lactation, the mammary gland is remodeled to produce and secrete milk to foster offspring, which involves alterations in adipocyte content.^70^ On the one hand, it was shown that mature adipocytes interconvert into so‐called pink adipocytes, which are responsible for milk production during lactation.^71^ A recent paper challenged this view and suggested that alveolar epithelial cells are responsible for the secretion of milk^70^ and that adipocytes contained within the mammary gland shrink during lactation to provide space for the other cells and increase in size again by hypertrophy during mammary gland involution.^70^ Another study demonstrated that this might be due to dedifferentiation of mature adipocytes in the mammary gland, during pregnancy and lactation.^72^

### 3.5 Plasticity and Function of Brite/Beige Adipocytes

From these reports, it is evident that adipose tissues not only differ based on their anatomical location, but that within individual adipose tissue depots a high degree of heterogeneity can be observed, which seems to be dependent on environmental factors. This is furthermore exemplified by the dynamics of brite/beige adipocytes, which appear upon cold stimulation within mainly white adipose tissue depots.^73^ We and others have shown that these cells arise from both de novo adipogenesis and interconversion from cells that resemble a white adipocyte and that upon warm adaption most of the brite/beige adipocytes acquire a white adipocyte‐like state.^26^, ^74^ Another case‐in point supporting the idea of adipocyte heterogeneity are recent reports that brite/beige adipocytes can utilize other thermogenic mechanisms besides UCP1 to dissipate heat.^75^ Thus, it was suggested that mainly brite/beige adipocytes in comparison to brown utilize futile calcium or creatine cycling to fuel thermogenesis.^76^, ^77^ Similarly, it was recently shown that based on external signaling cues brite/beige adipocytes with different functions can arise during development^78^, ^79^ again supporting the notion that adipose tissue is a highly plastic and dynamic organ that can react both during development as well as during adulthood to environmental stimuli to adapt to different metabolic requirements. Whether this concept is applicable to all adipocytes or whether just a subset of these cells has the above‐mentioned functionality remains to be shown.

Along with its role in energy and temperature homeostasis, adipose tissue has been recognized as an important endocrine organ. LEP was the first adipocyte‐derived hormone identified that primarily regulates food intake through promoting satiety.^80^ Individuals with impaired LEP gene expression show an obese phenotype, which can be reversed upon restoring leptin signaling. Adipose tissue releases a wide spectra of hormones and cytokines, such as TNF‐α^81^ and IL‐6, which control insulin sensitivity and inflammatory processes, or adiponectin that is involved in the regulation of glucose and fatty acids metabolism.^82^, ^83^, ^84^

## 4 Epigenetic Mechanisms

**Figure 4:**
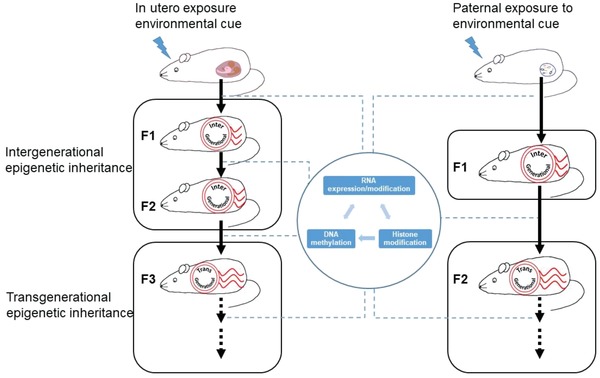
Environmental cues could be transmitted cross generations. In the case of in utero expose, F1 and F2 transmissions are classified as intergenerational epigenetic inheritance, F3 and further generations are transgenerational epigenetic inheritance. In the case of paternal exposure, F1 is classified as inter‐, while F2 and beyond as transgenerational epigenetic inheritance. DNA methylation, histone modification and s‐ and lncRNAs are involved in the epigenetic transmission.

Although DNA conveys the majority of the heritable information to subsequent generations, accumulating evidence demonstrates that epigenetic features are indeed passed to subsequent generations. Epigenetics hereby refers to heritable and functional changes to the genome that do not alter the nucleotide sequence itself.^85^, ^86^ This includes several different mechanisms, such as DNA methylation,^87^ histone modifications,^87^ higher order chromatin conformation,^88^, ^89^ small and long noncoding RNAs (s‐ and lncRNAs),^90^ as well as prions^91^ as illustrated in **Figure** **4**.

### 4.1 Inter‐ and Transgenerational Epigenetic Inheritance

In particular, it is important to distinguish between parental effects or intergenerational epigenetic inheritance and true transgenerational epigenetic inheritance.^92^ The later one requires that epigenetic traits are transmitted independent of repeated germline or developmental exposures and, as such, are stably passed on for more than three generations maternally or two generations paternally (Figure 4). In contrast, intergenerational epigenetic inheritance describes the phenomenon that parental or in‐utero exposures (including those of the developing germline in the embryo) result in novel acquired epigenetic traits, which can subsequently affect the health and biology of the offspring but are not passed on independently of the direct exposure.

Transgenerational epigenetic inheritance has been well documented in plants^93^, ^94^ and may have evolved to allow nonmobile organisms and their offspring to adopt rapidly to the immediate environment. In contrast, the existence and function of true transgenerational epigenetic inheritance in mammals are less well established. One reason for this is that several mechanisms exist in mammals, which limit the transmission of epigenetic signatures from one generation to the next. First, germ cells, which would be the carrier of the new epigenetic information, are separated from somatic cells by the so‐called Weismann barrier. Second, global epigenetic resetting during germline development^95^ and following fertilization^96^ represents a very efficient system to remove pre‐existing epigenetic modifications.

### 4.2 Epigenetic Modifications

Nevertheless, several reports have demonstrated that various types of epigenetic information can be transmitted as illustrated in Figure 4. Thus, it was shown that the part of the paternal DNA methylome zebra fish^97^ and in the mammalian embryo^98^ is inherited. Furthermore, it was demonstrated that some of the histone modifications from the paternal allele could be transmit to offspring.^99^, ^100^ Lastly, sncRNAs have been shown to convey the epigenetic information for the next generation,^101^, ^102^, ^103^ suggesting that epigenetic mechanisms could be in part responsible for the transmission of traits to the next or even multiple generations.

In conclusion, subsequent work will have to delineate, whether these reports of transgenerational epigenetic inheritance in mammals are due to a robust long‐term memory in other similar settings and what the role and function of this inheritance plays in the context of mammalian evolution.

## 5 Environmental Cues and the Development of Obesity

In recent years, numerous studies have been published, which demonstrate an influence of environmental exposure on subsequent generations, pointing toward a strong interplay of genetic and environmental factors.^104^ Thus, environmental cues can induce long lasting physiological changes in the progeny, and these acquired traits can be transmitted from parents to subsequent generations through nongenomic mechanisms.^105^, ^106^

### 5.1 Human Studies of Fetal Programming

Today it is accepted that environmental challenges at critical periods during development can have lifelong consequences for the health of the offspring. This process is known as fetal programming^107^ and extensive evidence exists demonstrating its importance in the propensity of the offspring to become obese.^108^, ^109^, ^110^ With respect to nutritional insults during development, epidemiological studies in humans support the conclusion that either under‐ or overnutrition of the mother during pregnancy will have profound and long‐term implications for the health of the offspring in adulthood, such as increased risk of developing adult obesity and the metabolic syndrome.^107^, ^111^ This is exemplified by the famous study of the Dutch famine, which demonstrates that undernutrition during gestation is associated with reduced glucose tolerance and increased insulin concentrations in the offspring.^112^ Similarly, higher maternal BMI is associated with increased type 2 diabetes in the offspring^113^ and maternal BMI is correlated to new born fat mass and fat ratio.^114^ Besides this, maternal triceps skinfold is associated with increased neonatal body fat,^115^ and it was shown that maternal hyperglycemia is associated with increased body fat in the offspring.^116^ This correlation involves mainly increased neonatal adipose tissue mass and not lean body mass, suggesting a link between programming and the capacity to form adipose tissue rather than overall growth.^117^ As expected, it is similarly established that increased maternal prepregnancy BMI and gestational weight gain is in turn associated with higher risk of cardiometabolic diseases in offspring,^118^ most likely as a secondary consequence of obesity.

It is important to note that correlations between fetal programming and metabolic health of the offspring are stronger in human epidemiological studies of undernutrition than overnutrition, because of the heavy confounding effects of prenatal and postnatal environment, in the case of the latter. In the studies investigating the role of overnutrition, exposure takes place throughout pregnancy and childhood, thus, the child is exposed and influenced by the food preferences of the mother, making it impossible to establish a specific link independent of acquired behavior (Figure 2). This notion is supported by a study on monozygotic twins, which demonstrates that even with highly similar DNA sequences, individuals can develop different degrees of adiposity and metabolic profiles.^119^ Further studies have revealed that in monozygotic twins with varying metabolic profiles, global DNA methylation patterns are changed,^120^ and specifically, the serotonin receptor promoter region is differentially methylated,^121^ suggesting that the postdevelopmental period can similarly affect obesity development. It should be noted however, that these changes could be the result of metabolic alterations rather than the cause.

In conclusion, the accumulated body of evidence suggests that in the context of humans, the environment can actively interfere in the programing of an individual leading to alterations in systemic metabolism.

### 5.2 Rodent Studies of Fetal Programing

As the different exposures that lead to the offspring's phenotype are difficult to separate multiple animal models have been utilized to study the mechanisms of fetal programming. To date, different environmental insults in rodents, such as, social stress,^122^, ^123^, ^124^ toxins,^125^, ^126^ infections,^127^ drugs of abuse,^128^, ^129^ undernutrition,^130^, ^131^ and overnutrition^108^, ^132^, ^133^, ^134^, ^135^, ^136^, ^137^, ^138^ have shown to play potential roles in the transmission of altered metabolic traits to the offspring. Especially in the context of in utero exposure, many studies have reported insults independent of paternal behavior, which could lead to changes in the progeny. Thus it was shown that maternal obesity leads to increased body weight and reduced insulin sensitivity in the offspring^139^ and similarly that maternal obesity can affect adipokine gene expression and secretion thus altering systemic metabolism. Furthermore, maternal obesity was shown to lead to reduced thermogenic capacity^140^ by changing the adipose tissue composition and functionality. These studies demonstrate a link between environmental cues and adipose tissue function.

In addition, multiple studies have implicated the CNS as a target for environmental cues. In this context, one study suggested a link between maternal obesity and brain inflammation^141^ as well as other cognitive and neurochemical abnormalities.^135^, ^137^, ^138^ One important study could show that maternal high‐fat diet feeding especially during lactation predisposes the offspring for obesity and impaired glucose homeostasis in mice, which is associated with an impairment of the hypothalamic melanocortin circuitry possibly due to an altered formation of POMC and Agouti‐related protein (AgRP) projections to hypothalamic target sites.^142^ While the mode of epigenetic transmission remained unclear the authors of this study could demonstrate that the main effector of this signal was insulin signaling in POMC neurons. In contrast, we have shown that maternal obesity can affect the dopaminergic circuity of offspring's thereby modulating food preference, which ultimately can lead to the development of obesity.^136^, ^143^, ^144^ Taken together these data point to an important role of the CNS as a target for epigenetic transmission of environmental cues especially during fetal development.

It should be noted that the above‐mentioned studies, while clearly demonstrating the effects of environmental cues on the progeny have several problems which makes them difficult to interpret. First, maternal behavior can play an important role in the transmission of an environmental cue. Therefore, all studies which study in utero or post‐weaning exposure require cross‐foster controls to exclude such effects and/or should employ in vitro fertilization.^136^, ^142^ Second, the changes observed in the offspring could occur independent of epigenetic alterations through modulation of developmental pathways and thus would not be strictly considered an epigenetic transmission.

### 5.3 The Paternal Lineage in Fetal Programming

These points can in part be overcome by studying the paternal lineage as transmitter of environmental cues. Especially in rodent studies, paternal influences on behavior which could in turn drive phenotypes in the offspring can be excluded by removal of the male from the breeding directly after conception. Furthermore, paternal transmission of an environmental cue could be considered an epigenetic transmission as the sperm in the father has to be altered and a direct effect of the environmental cue affecting developmental changes in utero can be excluded in such a model system. In this context it was shown already in 2014 in Drosophila that paternal high sugar intake can cause obesity in the offspring.^145^ Similarly, it was demonstrated that paternal high fat diet can lead to β‐cell dysfunction in female rat offspring.^146^, ^147^ It was reported that paternal HFD exposure induced the obesogenic phenotype in a rodent model system.^148^ Thus, progenies sired from an HFD exposed fathers were heavier than their controls, had the greatest adiposity and had the greatest concentration of serum cholesterol, triglyceride, high‐density lipoprotein, and nonesterified free fatty acids compared with chow diet sired littermates. Similarly, it was shown that paternal low‐protein diet was shown to lead to an increased cholesterol synthesis.^149^

Other studies have reported a higher propensity for obesity in the offspring following paternal hyperglycemia possibly by changes in the amount of brown fat^150^, ^151^ as well as in the growth hormone signaling axis. In this context, we could show recently that paternal cold exposure leads to hyperactive BAT formation thus protecting the offspring from obesity.^152^

Based on these data, it is evident that environmental cues can be transmitted through sperm possibly via epigenetic alterations. Interestingly, in contrast to maternal exposure the systemic targets of this exposure seem to be less defined and less CNS mediated, which could point to differences in mechanism regarding the transmission of these cues.

### 6 Effects of Environmental Cues Contributing to the Development of Obesity Across Multiple Generations

As discussed above, it is well established from both human and animal studies that the effects of parental dietary insults early in development can predispose the progeny to develop metabolic disorders. The majority of these studies so far concentrated on intergenerational effects of environmental exposure. However, to date, there are multiple studies investigating the effects of parental overnutrition across multiple generations, suggesting the existence of transgenerational epigenetic mechanisms. Human epidemiological studies suggest that grandparental overnutrition increases the rates of diabetes and cardiovascular disease risk in second‐generation offspring^153^ and that the origin of these effects is attributed to be more “grandpaternal” rather than “grandmaternal.” Another study reported that an increased risk for obesity was observed in children whose parents were of normal weight, but whose grandparents were obese.^154^

### 6.1 Transgenerational Effects of Parental Exposure on the Fetus

In comparison to human studies, experimental animal research has made tremendous progress in exploring the transmission of maternal overnutrition induced obesity and altered metabolic phenotypes up to the second generation. Interestingly, however, animal studies on maternal and/or paternal overnutrition report different phenotypes as well as differences in severity of the effects in the offspring. This is not surprising, since these effects are probably highly variable depending on the organism, strain, type of diet, length of exposure, which either induced a metabolic phenotype in the mother or father (i.e., obesity, hyperinsulinemia, and diabetes, etc.). A comprehensive overview of the different studies as well as the measured parameters can be found in **Table** **1**. Animal studies of paternal transmission have provided evidence that the carrier of environmental information occurs through changes in sperm. However, it is still unknown whether this transmission is stable in the adult and can be transmitted across multiple generations as suggested from human epidemiological studies. In this context, animal models have been essential in understanding the impact of a nutritional insult early in development and the risk of metabolic diseases in subsequent generations. These changes in modifications in sperm‐born factors due to nutritional insults are essential for establishing stable epigenetic marks and will provide means to identify a potential molecular basis to explain the transmission of developmental plasticity across generations. We will focus in this section only on the contribution of epigenetic heritability following maternal and/or paternal HFD exposure.

**Table 1:** Summary of the effect of maternal or paternal high‐fat diet (HFD) exposure or overfeeding and the metabolic traits on subsequent generations [F2 (second generation) and/or F3 (third generation)] in rodents. BW, body weight; BL, body length; GTT, glucose tolerance; ITT, insulin tolerance test, Chol, cholesterol, BG, blood glucose levels, IL, insulin levels; pancreatic and duodenal homeobox factor‐1 (Pdx1), neurogenic differentiation 1 (NeuroD1). F, female, M, male

Study	Maternal/paternal HFD exposure	Metabolic traits	Epigenetic mark	Sex	Generations	True epigenetic inheritance?	References
Sarker et al., 2018	Maternal, paternal	F2: ↑ BW (F,M), ↑ adipocity, ↓ ITT, ↑ Chol, ↑ IL (F,M)	No changes in sperm CpG methylation	F, M	F2, F3	Yes	144
		F3: ↑ BW (M), ↑ adipocity, ↓ ITT, ↑ Chol, ↑ IL (M)					
Sarker et al., 2019	Paternal	F2: ↑ BW (F,M), ↑ adipocity, ↓ ITT, ↑ Chol, ↑ IL (F,M)	↑ sperm tsRNAs, predominantly 5' tRNA halves	F, M	F2	Yes	Unpublished
Huypens et al., 2016	Maternal	F2: ↑ BW		F, M	F2	No	155
King et al., 2013	Maternal	F2: ↑ adiposity (M), ↓ IL, ↓ Chol		F, M	F2	No	156
Chambers et al., 2016	Maternal, paternal	F2: ↑ adiposity, ↑ Leptin	No changes in the F0 intratesticular GC transcriptome	M	F2	Yes	157
Huang et al., 2017	Maternal	F2: ↑ BW, ↓ GTT, ↓ β‐cell, ↓ Pdx1, ↓ NeuroD1		F, M		No	158
Gniuli et al., 2008	Maternal	F2: ↑ BW		M	F2	No	159
Pentinat et al., 2010	Maternal, paternal	F2: fasting hyperglycemia, ↓ GTT		M	F2	Yes	160
Fullston et al., 2013	Paternal	F2: ↑ BW		F, M	F2	Yes	133
Masuyama et al., 2016	Paternal	F2: ↑ BW, ↓ ITT, ↓ GTT, hypertention, ↑ Leptin, ↓ adiponectin (F,M)	Epigenetic modifications of the genes encoding adipocytokines adiponectin and leptin	F, M	F2, F3	Yes	161
		F3: ↑ BW, ↓ ITT, ↓ GTT, hypertention, ↑ Leptin, ↓ adiponectin (F,M)					
Hanafi et al., 2015	Maternal	F2: ↑ BG, BW	Alterations in sperm microRNA, ↓ global methylation of germ cell DNA	F, M	F2	No	162
Dunn et al., 2009	Maternal	F2: ↑ BL (F,M), ↓ ITT	Hypomethylation in GHSR CpG island (hypothalamus)	F, M	F2	No	163
Dunn et al., 2011	Maternal, paternal	F3: ↑ BL (F), ↑ GTT (M)	Changes in imprinted gene profile (liver)	F, M	F2, F3	Yes	132
de Castro Barbosa et al., 2015	Paternal	F2: ↑ BW	DNA methylation and miRNA in F0 sperm	F	F2	Yes	135
Wei et al., 2014	Paternal	F2:↓ ITT ↓ IL	DNA methylation in F0 sperm and F1, F2 pancreas	F,M	F2	Yes	164

### 6.2 Transgenerational Paternal Transmission of Physiological Changes

Investigations of the epigenetic inheritance via the paternal lineage as alluded to above, has the advantage of avoiding possible maternal or in utero confounding factors.^165^ Several rodent models of paternal HFD exposure introduced before conception affect the metabolic state of the offspring through epigenetic inheritance. For example, Mansuyama and co‐workers^161^ observed that paternal HFD prior to conception induced a metabolic syndrome‐like phenomenon including weight and fat gain, glucose intolerance, hypertriglyceridemia, abnormal adipocytokine levels, hypertension, and adiponectin and LEP gene expression and epigenetic changes over multiple generations. The authors found that the effect of paternal HFD consumption was weaker than that of maternal HFD exposure. Interestingly, when male offspring consumed normal laboratory chow for two generations, it completely abolished the effects of paternal HFD consumption on the offspring. Fullston et al. showed that paternal obesity initiated intergenerational transmission of obesity and insulin resistance in two generations of offspring.^133^ Pentinat et al. reported that neonatal overfeeding (induced by culling offspring to four pups during lactation) induced in first‐generation offspring fed and fasting hyperinsulinemia, hypertriglyceridemia, insulin resistance, and glucose intolerance, but not obesity.^160^ In contrast, second‐generation offspring showed a more moderate phenotype. The impaired glucose tolerance in first‐ and second‐generation offspring was attributed to peripheral insulin resistance, since β‐cell function remained normal or even increased in these offspring. Dunn and Bale have shown that maternal HFD increased body weight and body length phenotypes, which were persistent up to the third generation in female offspring conceived through the paternal lineage.^132^, ^163^ Further, an altered expression of the growth hormone receptors (GHSR) and associated DNA hypomethylation at the GHSR CpG island in the arcuate nucleus of hypothalamus was suggested to be a candidate for the intergenerational transmission of the increased body length phenotype.^132^

### 6.3 Mechanisms of Transgenerational Inheritance

We have shown recently that offspring born to maternal HFD exposed mothers developed obesity and insulin resistance up to the third generation in the absence of any further HFD exposure. This implicates that the male germ line is a major player in transferring this metabolic trait.^144^ However, methylome profiling of first‐ and second‐generation sperm revealed no significant difference between the offspring groups. This implies that the sperm methylome might not be the major carrier for the transmission of the phenotypes observed. In a follow‐up study, we studied, whether other epigenetic carriers could explain the strong metabolic phenotype observed in second‐ and third‐generation maternal HFD offspring (Sarker et al., unpublished data). Therefore, we investigated whether ncRNAs may play a crucial role in this transgenerational epigenetic inheritance and we could identify sperm tRNA‐derived small RNAs (tsRNAs) as a potential mediator for the transgenerational inheritance of maternal HFD induced obesogenic phenotypes in second‐generation offspring.

Based on the studies discussed above, it is plausible that environmental cues can be transmitted through epigenetic intergenerational and transgenerational mechanisms and thus impact metabolic control. However, the exact nature of these signals as well as the target organs that are affected by these processes is less well defined. As discussed above, the main mechanisms of epigenetic transmission are considered to be DNA methylation, histone modifications, and ncRNAs.^92^ A large host of studies have reported changes in DNA methylation patterns and histone modifications, however due to technical limitations it is difficult to infer a causal relationship between these two processes.^166^

Terashima et al.^167^ could show in an obese mouse model that the hepatic mRNA level of 7 genes was significantly altered in HFD male offspring compared to control mice. Interestingly, they could not detect changes in DNA methylation associated with HFD but differential histone H3‐occupancy at genes involved in the regulation of embryogenesis.

One exception in this context, are sncRNAs as these can be queried for their causal involvement in the transmission of epigenetic information.^101^, ^102^ In this context, it was reported that microinjection of sperm sncRNAs from traumatized mice or HFD‐fed mice leads to altered stress reactivity and metabolic phenotypes in the offspring.^124^, ^168^ SncRNAs can be divided into multiple classes namely, microRNAs (miRNAs), PIWI interacting RNAs (piRNAs), and tsRNAs, which in turn directly affect all levels of cellular function including other epigenetic marks such as DNA methylation,^169^, ^170^ making them an ideal target for the transmission of epigenetic marks. In the context of obesity, it was recently reported that changes in the paternal diet affects sperm tsRNAs levels. Similarly, it was shown that sperm derived tsRNAs if added through microinjection to the developing embryo are required and sufficient to confer the paternal information and elicit the same phenotype in the offspring.^101^, ^171^

## 7 Epigenetic Regulation of Adipose Tissue Plasticity and Function

The adipose tissue can be considered an interesting mediator of environmental cues for many reasons. First, in contrast to many other organs the adipose organ is highly plastic both at the developmental stage as well as during adulthood. Second, it has the capacity to form both energy storing as well as energy wasting cells with varying functionalities making it uniquely capable for reacting to environmental cues. Third, the adipose organ is tightly integrated with whole body energy homeostasis through adipokine signaling and fourth the long half‐life of an adipocyte can confer a stable response to certain cues.^34^

Thus, in particular epigenetic mechanisms are expected to be of key relevance for adipose tissue development and remodeling,^172^, ^173^ possibly due to the key characteristic of the epigenome as dynamic integrator and memory of environmental and metabolic changes.^174^ Importantly in this context, pharmacological modulation of the epigenome has been recognized as promising new treatment strategy and several drugs are already in clinical studies targeting various diseases.^175^ While much of our current molecular and epigenetic understanding of adipocyte specification is based on in vitro cell culture differentiation systems,^176^, ^177^, ^178^, ^179^, ^180^, ^181^, ^182^ recent work has shown epigenetic differences between adipocytes in vivo and in vitro,^183^ which emphasize the importance to establish a comprehensive molecular understanding of adipose tissue biology based on in vivo datasets. In this context, the different windows of susceptibility of adipose tissue in mice versus humans have to be taken into consideration (see also Section 3.2). While mouse adipose tissue develops mainly in the postnatal period,^33^, ^35^ which coincides with the findings of Vogt et al. who demonstrate that the effect of maternal high‐fat feeding on neuronal insulin sensitivity is most critical during the weaning period,^142^ the human critical period for adipose tissue development is possibly during gestation.^33^, ^35^

Another critical point that has to be kept in mind is sexually dimorphic effects of parental insults on adipose tissue development. Thus it was shown that the effects of maternal nutrient reduction on expression of genes regulating cortisol metabolism and adipogenic genes in fetal baboon adipose and liver tissues followed a sexually dimorphic pattern^184^, ^185^ while a dimorphic acceleration of pericardial, subcutaneous, and plasma lipid increase was observed.^186^ Furthermore, maternal undernutrition during gestation and weaning resulted in sex‐specific effects on postnatal adiposity and related metabolic profiles in adult rat offspring.^187^, ^188^

### 7.1 DNA Methylation Changes

Recent work has suggested a key role for epigenetic mechanisms in the regulation of adipose tissue development.^173^ In vitro studies have suggested a role for DNA methylation in the development of adipocytes, i.e., inhibition of DNA methylation induced the differentiation toward the adipose lineage.^176^, ^177^ Genome‐wide analyses have similarly reported DNA methylation changes during in vitro adipocyte differentiation^179^ and between in vitro white and brown adipocyte precursors.^189^

In human studies it was shown by Dahlman et al.^190^ that the adipocyte epigenetic signature in obese women, who lost weight postbariatric surgery is characterized by global hypomethylation and differential DNA methylation of multiple adipogenesis genes. These data are supported by another study, which demonstrated that obesity genes are differentially modified in patients before and after weight loss through bariatric surgery.^191^ Similarly, it was shown that adiposity is associated with alterations in the adipose tissue DNA methylation profile in adipose tissue.^192^ These data demonstrate that epigenetic changes can occur in adipose tissue in response to environmental alterations, however if these changes are causative for functional differences and whether these changes can be transmitted toward future generations cannot be deuced from this work.

Several studies using rodent model systems have analyzed the epigenetic alterations in the context of inter‐ and transgenerational models. Thus, it was reported that maternal obesity can alter the propensity of white adipose tissue to form new adipocytes and that these changes were associated with alterations of DNA methylation in the progeny.^193^ Another study reported that maternal obesity led to extensive changes in lipogenic and glucose transport genes in WAT, suggesting sensitization to insulin signaling. Furthermore, offspring showed an induction of genes associated with adipogenic genes. These transcriptomic changes were associated with alterations in DNA methylation of CpG sites, proximal to developmentally important genes suggesting that maternal obesity might alter adipocyte commitment and differentiation.^193^ In the context of adipocyte function, Masuyama et al. reported that HFD exposure in utero led to alterations in the methylation of the LEP and the adiponectin promoter, which was correlated to changes in gene expression of these two adipokines.^194^, ^195^ Similarly it was reported that the time window shortly after birth is an active period for epigenetic remodeling different adipose depots especially in the upstream enhancer, consistent with LEP induction during adipogenesis.^196^ Ding et al. reported that DNA methylation of inflammatory genes was altered up to an F2 generation in a model of in utero exposure and correlated with macrophage infiltration and inflammation of adipose tissue.^197^ Similar to the above‐mentioned human studies, these studies indicate that epigenetic mechanisms exist which could transmit an environmental insult possibly even across multiple generations, however further studies will be needed to address whether these observed epigenetic changes are the result or the actual drivers of the observed phenotypic alterations.

Novel work in the context of brown, brite/beige and white adipocytes suggests that these cells might be of relevance as functional mediators of epigenetic alterations. Interesting in this context is a study from Lim et al. which demonstrate that DNA methylation is a stable epigenetic signature for brown and white cell lineage before, during, and after differentiation.^198^ The authors identified 31 genes, whose promoters were differentially methylated between white and brown adipocytes. Most notably are changes in methylation of Hox family genes, which have been implicated as markers for white and brown adipocytes.^199^ These data suggest that the DNA methylome of the different adipocyte subtypes is highly variable and dependent on environmental cues and can pre‐program cellular functionality.

### 7.2 Histone Modifications

Equally a number of histone modifiers have been shown to be involved in the regulation of BAT, e.g., histone deacetylases,^178^, ^181^, ^200^ euchromatic histone lysine methyltransferase 1,^201^ or G9a.^182^ In a seminal study by Roh et al.,^183^ the authors employed cell‐type‐specific profiling in vivo, to study the temperature‐dependent epigenomic plasticity of the different adipocyte subtypes. They could show that despite a profound shift in cellular identity when brite/beige adipocytes in the inguinal depot reverted to white adipocytes these cells retain an epigenomic memory of prior cold exposure at various enhancers that allows the rapid expression of thermogenic genes in response to a temperature stimulus. In 2007, it was already shown by Kiskinis et al. that RIP140,^202^ which is a corepressor for nuclear receptors, and which represents an important hub to control energy homoeostasis is essential for both DNA and histone methylation to maintain gene repression of the Ucp1 gene, thereby regulating white adipocyte identity through methylation of specific CpG residues and histones. Similar data for the involvement of histone methylation in the maintenance of adipose tissue functionality were obtained from the study of the histone demethylase LSD1. In 2014, Chen et al.^203^ could show that LSD1 regulates the oxidative metabolism of white adipose tissue. This was substantiated by two subsequent studies, which demonstrated that LSD1 demethylates H3K9 on promoters of Wnt signaling components and the transcription factor Zfp516^204^ and thus regulates brown and brite/beige adipocyte development.^205^ Further evidence for the role of histone modification came from a study by Ohno et al.^201^ which identified EHMT1 as a H3K9 methyltransferase which controls brown cell fate. The authors demonstrated that loss of EHMT1 in brown adipocytes leads to H3K9 me2/3 demethylation and muscle cell differentiation, while enhanced EHMT1 expression induces a brown adipocyte thermogenic program again demonstrating the importance of epigenetic marks in the regulation of adipose tissue plasticity. Further studies identified also Jmjd3 as an important H3K9 methyltransferase, which promotes brown and brite/beige adipocyte formation and it was shown that Jmjd3‐mediated H3K27me3 dynamics can orchestrate brown adipose tissue development and white adipose tissue plasticity.^206^ Similarly, it was shown that histone‐lysine N‐methyltransferase 2D that is a H3K4 methyltransferase is required for brown cell differentiation,^207^ while targeted inactivation of Lysine N‐methyltransferase 2C was required for the regulation of white adipogenesis.^208^

### 7.3 Future Challenges

A large number of small RNAs have been implicated in the regulation of adipocyte formation and function and furthermore small RNAs have been shown to affect adipose tissue plasticity. As discussed above, the intergenerational transmission of obesogenic traits through the paternal lineage has been shown to be mediated through changes in the sperm tsRNAs. So far, however, no studies have directly implicated small RNAs in the transmission of epigenetic information from the parents to the offspring to modulate adipose tissue function of plasticity. Nevertheless, there are indications that such factors could act through changes in chromatin structure as it was reported that piRNAs and noncoding RNAs can affect the de novo DNA methylation of imprinted loci in mice. Similarly, it was reported that different small RNAs can associate with mediator complex to modulate chromatin architecture and transcription.^169^, ^209^ Clearly, further studies will be required to address these points in more details.

In summary, from these collected works, the notion can be derived that in addition to genetic variations within the population, transmission of environmental cues might influence energy metabolism in the offspring over several generations. Through the transmission of stable marks energy metabolism might be altered, which in turn can predispose an individual to become obese. Taking an adipocentric view, expansion of adipose tissue is often accompanied by changes within the epigenome of the highly dynamic adipose tissue. Similarly, many different model systems, which have been used to target the epigenetic machinery at different pathways (sncRNA, methylation, histone modification), have revealed that both function and plasticity of adipose tissue is highly dependent on these modifications.

From these deliberations several questions arise: First, it remains unclear (with very few exceptions), whether the transmitted environmental cues are the cause of altered adipocyte plasticity and functionality, thus contributing to changes in energy metabolism, or whether they constitute secondary modifications arising as a consequence of alterations in energy metabolism. Further studies on the relationship between these two aspects, utilizing novel (epi)genetic models and methods, will be required to understand and delineate the processes of epigenetic inheritance, adipose tissue plasticity, and function and alterations in energy metabolism.

Second, it remains unclear how any of the epigenetic alterations will ultimately modulate changes in adipose tissue development, plasticity, and function in the offspring. This holds true for inter‐ and transgenerational inheritance, in which epigenetic alterations might modulate developmental programs, thus affecting for example neuronal circuitry or other regulatory pathways.

Third, especially in the presented examples of putative transgenerational transmission of epigenetic marks, it is not understood, how changes are prorogated to subsequent generations. As discussed above this might involve developmental changes, which in turn affect sperm epigenetic modifications, which could explain certain observations such as more pronounced phenotypes in the F2 generation. To delineate this point novel tools will be needed to trace alterations across multiple generations to define cause and effect in the modulation of epigenetic marks.

## Conflict of Interest

The authors declare no conflict of interest.
